# Positive perceptions of electronic cigarettes relative to combustible cigarettes are associated with weaker support for endgame policies on combustible cigarettes: A population-based cross-sectional study in Hong Kong

**DOI:** 10.18332/tid/110697

**Published:** 2019-08-28

**Authors:** Yongda S. Wu, Man Ping Wang, Sai Yin Ho, Yee Tak Cheung, Antonio Kwong, Vienna Lai, Tai Hing Lam

**Affiliations:** 1 School of Nursing, The University of Hong Kong, Pokfulam, Hong Kong; 2 School of Public Health, The University of Hong Kong, Pokfulam, Hong Kong; 3 Hong Kong Council on Smoking and Health, Wan Chai, Hong Kong

**Keywords:** electronic cigarettes, tobacco endgame, policy, smoking renormalization

## Abstract

**INTRODUCTION:**

Positive perceptions of electronic cigarettes (e-cigarettes) relative to combustible cigarettes (CCs) may erode support for endgame policies on CCs through smoking renormalization (increasing public acceptance of smoking). We investigated the associations between perceptions of e-cigarettes relative to CCs and support for endgame policies on CCs in Hong Kong.

**METHODS:**

Adult respondents (N=2004) were surveyed using landline random digit dialing in 2015. Perceived relative harm and relative addictiveness of e-cigarettes were combined as an overall perception of e-cigarettes relative to CCs with 5 levels and we analyzed individually ‘neutral/positive/mixed/unknown’ perceptions against the ‘negative’ perception. Five individual items with dichotomous responses assessed the support for endgame policies on CCs. Support for banning the sale/use of CCs (yes/no) was also assessed. Multivariable regressions yielded adjusted odds ratios (AORs) of supporting endgame policies (individual policy items, all 5 policy items, at least 1 policy item, banning the sale/use of CCs) in relation to perceptions of e-cigarettes relative to CCs, adjusting for age, education attainment, marital status, CC smoking status and ever e-cigarette use.

**RESULTS:**

Support for individual endgame policy items (from 51.8% to 80.0%), banning the sale (63.8%) and use (67.5%) of CCs were generally high. Few respondents perceived e-cigarettes as more harmful (16.6%) or more addictive (9.3%) than CCs. Positive perceptions of e-cigarettes (24.0%) were associated with less support for ‘ban CC sales in 10 years if there is a product providing nicotine not made from tobacco’ (AOR=0.62, 95% CI: 0.40–0.97), ‘ban CC use when it’s prevalence falls below 5%’ (AOR=0.66, 95% CI: 0.44–0.98) and ‘banning the sale of CCs’ (AOR=0.63, 95% CI: 0.42–0.94).

**CONCLUSIONS:**

Positive perceptions of e-cigarettes relative to CCs were associated with less support for endgame policies on CCs in Hong Kong. Public health actions are needed to disseminate evidence-based knowledge of e-cigarettes.

## INTRODUCTION

Electronic cigarettes (e-cigarettes) are increasingly popular in Hong Kong, US and European countries^1-3^ due to massive advertising and promotion^2-4^. Many e-cigarette promotions claim that these products are free from toxins and less addictive than combustible cigarettes (CCs)^5^. Such promotions of e-cigarettes ignore the fact that an increasing number of studies have found respiratory toxicants, irritants and similar levels of nicotine in aerosols from e-cigarettes compared to CCs^6-9^. E-cigarette promotions with health claims have been linked to positive perceptions of e-cigarettes^10^, which has led to less support for restrictions on e-cigarettes, e.g. use and advertising^11,12^. Public support is crucial for the implementation and enforcement of tobacco control (TC) policies^13^ including those on e-cigarettes.

E-cigarette regulations in Hong Kong are less stringent than for CCs. Although use of e-cigarettes in smoke-free areas is prohibited, only those with nicotine need to be registered as a pharmaceutical product and obtain permission for sale (none has done so as of February 2019), and nicotine-free e-cigarettes are under no other specific regulation. Most of the e-cigarettes in Hong Kong claim to be nicotine-free and can be marketed freely in retail stores and online, and are often promoted as a healthier tobacco product^14^. Over 80% of the Hong Kong population is aware of e-cigarettes^15^, nearly one-third perceives e-cigarettes as less harmful than CCs and such perceptions are associated with reduced support for e-cigarette regulations^16^.

Positive perceptions of e-cigarettes may renormalize smoking, i.e. e-cigarettes may increase the attractiveness of smoking^17^. If e-cigarettes are portrayed as a healthier tobacco product this might translate to the personal experience and public performance of using CCs^17,18^. Smoking has become less acceptable to the public as control policies (e.g. increased smoke-free areas) become more stringent and the harms of CCs (the predominantly used tobacco product) are more well known^19^. Effective dissemination of the harms of CC use increases public support for more stringent control policies^20^. In contrast, increasing e-cigarette use and promotions may increase public acceptance of smoking, which in turn may erode support for further control policies on CCs (e.g. endgame policies on CC use). Studies on this renormalization hypothesis are few and have focused on whether e-cigarette use leads to initiation of CC use in youth^21^. A recent Surgeon General’s report^22^ and a meta-analysis^23^ concluded that e-cigarette use was associated with the uptake of CCs. No study has investigated the effect of renormalization by e-cigarettes on control policies towards CCs.

Endgame policy is a unique type of TC policy that refers to an end to tobacco use. But consensus on the implementation of endgame is lacking and various approaches have been proposed^24,25^. In Hong Kong, tobacco endgame is generally referred to as the elimination of CC use since combustible cigarettes are the predominantly used tobacco products at present^26^. Over 70% of respondents in a 2012 population-based survey supported banning tobacco sale, possession or use^27^. Local tobacco advocates have urged the government to strengthen TC policies towards a CC smoking prevalence of 5% or lower in 2027 and to end CC use and sale^28^.

The relation between positive perceptions of e-cigarettes and public support for control policies on CCs remains unclear. We investigated the cross-sectional association of perceptions of e-cigarettes with support for tobacco endgame policies on CC use in a population-based survey.

## METHODS

### Study design

We analyzed data from the Tobacco Control Policy-related Survey 2015, funded by the Hong Kong Council on Smoking and Health (COSH) with the consultancy of the Schools of Nursing and Public Health, University of Hong Kong. The fieldwork was conducted by the Public Opinion Program, University of Hong Kong, a well-known local survey agency. Details of the survey have been published elsewhere^16^. In brief, 5252 Chinese speaking respondents from Hong Kong, aged 15 years or older were interviewed, including 1706 current CC users (current smokers, consumed at least 1 CC in the past 7 days), 1712 past CC users (ex-smokers), and 1834 never smokers, with a response rate of 17.8% (over 6071 eligible cases and 23497 cases whose survey was rescheduled but not done within the survey period). They were sampled through a two-stage landline random digit dialing. The sampling telephone number list was based on a residential telephone directory. Adding 1 or 2 to, or subtracting 1 or 2 from the last digit of the telephone numbers from the directory allowed the inclusion of unlisted numbers. One respondent was selected, using the ‘nearest birthday’ method, from eligible family members in a successfully contacted household. Current smokers and ex-smokers were over-sampled to obtain reliable estimates since their prevalence was low in Hong Kong (10.5% for current smokers and 5.6% for ex-smokers in 2015)^26^. To collect respondents’ opinions on various tobacco control policies and avoid a lengthy questionnaire that might have jeopardized data quality, a random sub-sample of respondents was selected to obtain opinions on endgame policies for CCs. Among the 4517 (of 5252) respondents who were aware of e-cigarettes and reported their perceptions of e-cigarettes, 2004 respondents who also reported their opinion of endgame policies on CCs were included in this study. Ethical approval of the survey was granted by the Institutional Review Board of the University of Hong Kong/Hospital Authority Hong Kong West Cluster (Ref: UW15-108). All methods were performed in accordance with the relevant guidelines and regulations. All respondents gave informed consent before the survey started.

### Measurements

Perceived relative harm of e-cigarettes (vs CCs) was assessed with 6 response items: ‘much more harmful’, ‘slightly more harmful’, ‘similar’, ‘slightly less harmful’, ‘much less harmful’, and ‘I don’t know’. The responses were combined into 4 categories (‘more/slightly more harmful’, ‘similarly harmful’, ‘less/slightly less harmful’, and ‘I don’t know’) for analysis due to a small number of observations in some of the responses. Perceived relative addictiveness of e-cigarettes (vs CCs) was asked with 5 slightly different responses (‘more addictive’, ‘similar’, ‘less addictive’, and ‘not addictive’, and ‘I don’t know’) given that e-liquids could be free of nicotine. The ‘less addictive’ and ‘not addictive’ responses were also combined for analysis. We classified overall perceptions of e-cigarettes relative to CCs based on the perceived relative harm and addictiveness of e-cigarettes (Table 1). The ‘Neutral’ perception was both ‘similar’ responses to the questions on harm and addictiveness relative to CCs, and the ‘Unknown’ perception was both ‘I don’t know’ responses. The ‘Negative’ perception included the following types of responses: both ‘more harmful’ and ‘more addictive’, ‘more harmful’ and ‘similar addictiveness’, and ‘similar harm’ and ‘more addictive’. The ‘Positive’ perception was defined in a similar manner (both ‘less harmful’ and ‘less addictive’, ‘less harmful’ and ‘similar addictiveness’, and ‘similar harm’ and ‘less addictive’). Other combinations of responses were grouped into ‘Mixed’ perception of e-cigarettes relative to CCs.

**Table 1:** Categorization of perceptions* of e-cigarettes relative to combustible cigarettes for analysis

*Perceive relative harm of e-cigarettes*	*Perceive relative addictiveness of e-cigarettes*			

	*More*	*Similar*	*Less*	*Don’t know*
More	Negative	Negative	Mixed	Mixed
Similar	Negative	Neutral	Positive	Mixed
Less	Mixed	Positive	Positive	Mixed
Don’t know	Mixed	Mixed	Mixed	Unknown

* In a representative sample (N=2004) of the Hong Kong adult population in 2015.

This study included 5 individual endgame policy items on CCs with dichotomous responses (yes/no): ‘ban CC sales in 10 years if there is a product providing nicotine not made from tobacco’, ‘ban people who are born after the year 2010 from smoking CCs’, ‘license to purchase CCs and to smoke’, ‘set a quota for CC retail and reduce it on a yearly basis’, and ‘ban CC use when its prevalence falls below 5%’. These items were analyzed as supporting all 5 policies, at least 1 policy as well as individual policies. ‘Refuse’ and ‘I don’t know’ responses to individual endgame policy items were excluded in respective analyses since they were few (ranging from 4.1% to 10.4%). Another 2 questions assessed support for 2 broad endgame policies on CCs: ‘Do you support completely banning the sale of CCs’, and ‘Do you support completely banning the use of CCs’. Both questions were asked with 7 ‘support’ responses with different deadlines (immediately, in 1 year, in 3 years, in 5 years, in 10 years, after 10 years, deadline not yet determined) and 6 ‘against’ responses with different reasons (people have a right to smoke, negative effect on the economy, ban is useless, increase CC smuggling, being a smoker, others). ‘Support’ and ‘against’ responses were combined respectively for analysis. Ever use of e-cigarettes was inquired and users were further asked usage in the past 30 days. But we only used the information on ever use due to the very few respondents with use in the 30 days. Respondents’ sociodemographic characteristics were also collected.

### Statistical analysis

Sociodemographic characteristics, perceptions of e-cigarettes relative to CCs and support for endgame policies on CCs were weighted by age, sex, and CC smoking status distributions of the general population in Hong Kong in 2015^26^. Multivariable logistic regressions yielded adjusted odds ratios (AORs) of support for the endgame policies on CCs (individual policy items, all policy items, and at least 1 policy item) in relation to perceptions of e-cigarettes relative to CCs, adjusting for age, education attainment, marital status, CC smoking status and ever e-cigarette use. Marital status was adjusted because married people were more likely to be concerned about the harms to their partners or children caused by smoking, which in turn may affect their perceptions of e-cigarettes and/or support for endgame policies on combustible cigarettes^29,30^. The relations between perceptions of e-cigarettes relative to CCs and support for 2 broad endgame policies were examined with a similar method to check the robustness of results of individual endgame policy items. As the associations might differ by CC smoking status, effect modification (ex-smokers, current smokers vs never smokers) was tested by adding product terms (perceptions of e-cigarettes relative to CCs × CC smoking status) in the model and checked by likelihood ratio test comparing models with or without the product terms. All analyses were conducted using Stata (Version 15.1, TX: StataCorp LP, College Station, TX, USA).

## RESULTS

Respondents reporting their support for endgame policies on CCs and those not included in the analysis shared similar sociodemographic characteristics, except that fewer included respondents had a monthly household income of HK$30000 or above (1US$=7.8HK$) (24.6% vs 27.8%, p=0.04, data not shown in tables). Over half of the respondents (52.2%) were female, and two-thirds (66.7%) were aged 30–39 years (Table 2). Ever e-cigarette use was 5 rare (1.1%) and 11.5% of respondents were current CC users. Age, highest educational attainment, marital status, CC use and ever e-cigarette use were associated with perceptions of e-cigarettes (all p≤0.05) and adjusted in logistic regressions.

**Table 2:** Respondents’ sociodemographics, use of e-cigarettes and their associations with perceptions of e-cigarettes relative to combustible cigarettes (CCs) (N=2004)

	*All respondents (%)*	*Perceptions of e-cigarettes relative to CCs (%)*					

		*Negative*	*Neutral*	*Positive*	*Mixed*	*Unknown*	p*
**Female**	52.2	39.3	57.1	56.4	49.7	54.8	0.066
**Age (years)**							**<0.001**
15–29	23.1	27.9	20.0	35.5	22.5	5.9	
30–39	66.7	64.5	71.6	57.3	66.4	78.4	
≥40	10.2	7.7	8.5	7.2	11.1	15.7	
**Highest educational attainment**							**0.001**
Primary or below	7.1	5.6	4.6	4.5	7.4	13.5	
Secondary	47.3	52.0	37.5	48.9	46.5	52.3	
Tertiary or above	45.7	42.4	57.9	46.6	46.2	34.2	
**Marital status**							**<0.001**
Single	32.9	36.3	33.1	45.2	30.7	16.9	
Married or cohabited	62.6	56.3	64.4	51.1	64.8	77.4	
Divorced or widowed	4.5	7.4	2.5	3.6	4.4	5.7	
**Monthly household income (HK$)**							0.110
less than 10000	9.0	6.9	5.1	10.3	9.2	11.6	
10000–19999	13.5	12.7	13.3	14.1	12.0	17.3	
20000–29999	18.1	25.1	8.8	20.0	19.1	17.0	
≥30000	59.4	55.3	72.8	55.5	59.8	54.0	
**Combustible cigarette (CC) use status**							**<0.001**
Never smokers	83.9	82.6	88.6	87.9	81.6	79.5	
Ex-smokers	4.7	3.5	4.1	3.6	5.3	6.2	
Current smokers	11.5	13.9	7.3	8.5	13.1	14.3	
**Ever e-cigarette use**	1.1	1.4	0.0	1.2	1.7	0.1	**0.050**

All analyses were weighted by age, sex, and CC smoking status distribution of the Hong Kong population in 2015.

* Based on chi-squared test.

Few respondents perceived e-cigarettes as more harmful (16.6%) or more addictive (9.3%) than CCs and about a quarter did not know the relative harm (25.4%) and addictiveness (27.7%) of e-cigarettes (Table 3). Only 9.5% held overall negative perceptions of e-cigarettes relative to CCs, while 24.0% held positive perceptions and 36.6% held mixed perceptions. Respondents’ support for individual endgame policy items on CCs was generally high (from 51.8% to 80.0%). Over one-quarter of the respondents (29.5%) supported all 5 individual policy items and 88.3% supported at least 1 policy item. Respondents’ support for broad endgame policies on CCs was also high: 63.8% for banning the sale of CCs, and 67.5% for banning the use of CCs.

**Table 3:** Perceptions of e-cigarettes relative to combustible cigarettes (CCs) and support for endgame policies on CCs (N=2004)

	%
**Perceptions of e-cigarettes relative to CCs**	
**Perceived relative harm of e-cigarettes**	
More harmful/slightly more harmful	16.6
Similarly harmful	22.7
Less harmful/slightly less harmful	35.4
Don’t know	25.4
**Perceived relative addictiveness of e-cigarettes**	
More addictive	13.8
Similarly addictive	33.4
Less addictive/not addictive	25.1
Don’t know	27.7
**Overall perceptions of e-cigarettes relative to CCs**	
Negative (more harmful/addictive)	9.5
Neutral (similarly harmful/addictive)	14.0
Positive (less harmful/addictive)	24.0
Mixed perceptions	36.6
Unknown (do not know both harm and addictiveness)	15.9
**Support for endgame policies on CCs**	
Ban CC sale in 10 years if there is a product providing nicotine not made from tobacco	70.5
Ban people who are born after the year 2010 from smoking CCs	51.8
License to purchase CCs and to smoke	54.1
Set a quota for CC retail and reduce it on a yearly basis	80.0
Ban CC use when its prevalence falls below 5%	62.1
All policies	29.5
At least 1 policy	88.3
Banning the sale of CCs	63.8
Banning the use of CCs	67.5

All analyses were weighted by age, sex, and CC smoking status distribution of the Hong Kong population in 2015.

Table 4 shows that positive perceptions of e-cigarettes relative to CCs are associated with less support for ‘ban CC sales in 10 years if there is a product providing nicotine not made from tobacco’ (AOR=0.62, 95% CI: 0.40–0.97), ‘ban CC use when it’s prevalence falls below 5%’ (AOR=0.66, 95% CI: 0.44–0.98) and ‘banning the sale of CCs’ (AOR=0.63, 95% CI: 0.42–0.94). A marginally significant association with ‘ban people who are born after the year 2010 from smoking CCs’ (AOR=0.71, 95% CI: 0.49–1.05, p=0.08) was also observed. ‘Unknown’ perceptions were associated with less support for each of the 5 individual policy items, all 5 policy items, at least 1 policy item and ‘banning the sale of CCs’ (all p<0.05). Product terms (perceptions of e-cigarettes relative to CCs × CC smoking status) used to test effect modification were not statistically significant (all p>0.05) and this was consistent with results from likelihood ratio tests (all p>0.05, suggesting adding product terms did not significantly improve model fit) (data not shown in tables).

**Table 4:** Association of perceptions of e-cigarettes relative to combustible cigarettes (CCs) with support for endgame policies on CCs (N=2004)

*Support for endgame policies on CCs*	*Perceptions of e-cigarettes relative to CCs (positive/neutral/mixed/unknown vs negative)*							

	*Crude OR (95% CI)*				*Adjusted OR (95% CI)^a^*			

	*Neutral*	*Positive*	*Mixed*	*Unknown*	*Neutral*	*Positive*	*Mixed*	*Unknown*
Ban CC sale in 10 years if there is a product providing nicotine not made from tobacco	0.57 (0.36–0.90)*	0.63 (0.41–0.96)*	0.59 (0.40–0.88)**	0.49 (0.32–0.74)***	0.55 (0.34–0.89)*	0.62 (0.40–0.97)*	0.56 (0.37–0.84)**	0.43 (0.28–0.67)***
Ban people who are born after the year 2010 from smoking CCs	0.71 (0.47–1.07)^†^	0.71 (0.49–1.02)^†^	0.81 (0.58–1.13)	0.70 (0.48–1.00)*	0.69 (0.45–1.05)^†^	0.71 (0.49–1.05)^†^	0.78 (0.55–1.10)	0.60 (0.41–0.88)**
License to purchase CCs and to smoke	0.85 (0.57–1.26)	0.95 (0.66–1.36)	0.85 (0.61–1.19)	0.58 (0.40–0.82)**	0.78 (0.51–1.19)	0.90 (0.61–1.32)	0.85 (0.60–1.20)	0.54 (0.37–0.79)**
Set a quota for CC retail and reduce it on a yearly basis	1.12 (0.71–1.77)	1.03 (0.68–1.55)	0.94 (0.64–1.37)	0.59 (0.40–0.88)**	0.98 (0.59–1.60)	0.90 (0.57–1.41)	0.92 (0.51–1.38)	0.57 (0.37–0.88)*
Ban CC use when its prevalence falls below 5%	0.80 (0.53–1.22)	0.66 (0.45–0.96)*	0.81 (0.57–1.16)	0.52 (0.36–0.75)***	0.77 (0.50–1.21)	0.66 (0.44–0.98)*	0.78 (0.54–1.13)	0.42 (0.28–0.63)***
All 5 policy items	0.89 (0.58–1.37)	0.86 (0.58–1.28)	0.83 (0.58–1.18)	0.67 (0.46–1.00)*	0.87 (0.56–1.37)	0.83 (0.55–1.24)	0.81 (0.55–1.17)	0.61 (0.40–0.92)*
At least 1 policy item	0.81 (0.44–1.47)	0.77 (0.44–1.33)	0.78 (0.47–1.29)	0.43 (0.26–0.73)**	0.74 (0.40–1.37)	0.76 (0.43–1.34)	0.76 (0.45–1.29)	0.40 (0.23–0.68)***
Banning the sale of CCs	0.77 (0.51–1.17)	0.63 (0.43–0.92)*	0.75 (0.53–1.07)	0.72 (0.49–1.05)^†^	0.72 (0.47–1.12)	0.63 (0.42–0.94)*	0.72 (0.50–1.04)^†^	0.62 (0.41–0.92)*
Banning the use of CCs	1.04 (0.69–1.57)	1.00 (0.69–1.46)	1.08 (0.77–1.52)	0.94 (0.65–1.37)	0.96 (0.62–1.49)	1.02 (0.68–1.52)	1.05 (0.73–1.51)	0.81 (0.54–1.20)

a Adjusting for age, highest educational attainment, marital status, ever e-cigarette use and CC smoking status (never smoker, ex-smoker, current smoker). In a representative sample (N=2004) of the Hong Kong adult population in 2015.

† p<0.1

* p<0.05

** p<0.01

*** p<0.001.

## DISCUSSION

We found positive perceptions of e-cigarettes relative to CCs were associated with less support for endgame policies on CCs. Associations were observed for individual endgame policy items and broad endgame policies, suggesting robustness in the results. These results were similar to those of previous studies, which found that more harmful or more addictive perceptions of e-cigarettes were associated with greater support for e-cigarette regulations on use, sale, and advertising^12,16^, and that perceptions of the negative health effects of smoking CCs were associated with supporting control policies on the use and sale of CCs^20,31^. Similar to the adult population of the US, only a small proportion (9.5%) of adults in Hong Kong held negative perceptions of e-cigarettes (perceived e-cigarettes as more harmful or more addictive than CCs)^32^. Uncommon negative perceptions of e-cigarettes relative to CCs could be partly attributed to massive e-cigarette marketing^4^ that featured harm reduction (such as no tar, no tobacco^5,33^). Since promotions of e-cigarettes may foster positive perceptions of the product, our findings suggest massive promotion of e-cigarettes may subsequently obstruct endgame to CCs by shrinking its public support.

Our findings are in favor of the renormalization hypothesis: positive perceptions of e-cigarettes relative to CCs may erode public support for control policies on CCs. One possible mechanism of our findings is that e-cigarettes may alter attitudes towards smoking^34^. E-cigarette use experience and performance closely mimic that of combustible cigarettes, which may empower it to shape a favorable attitude towards smoking in the public given the positive perceptions of e-cigarettes relative to CCs^18^. Further longitudinal studies and randomized control trials are needed to establish the causal relations between positive perceptions of e-cigarettes relative to CCs and reduced support for control policies on CCs. The underlying mechanism of smoking renormalization hypothesis, which would inform education and counter promotion campaigns on e-cigarettes, should also be carefully examined.

Our findings should also be interpreted in the context of ‘harm reduction’ strategy advocated by the tobacco companies, in which the health consequences of e-cigarettes are overlooked. The level of harm of e-cigarettes varies due to the diversity of products and how they are used, which decide the composition and amount of toxic substances in aerosols emitted^6^. The level of nicotine delivered by e-cigarettes could be similar to that from CCs^6,9^. Limited short-term evidence precludes the conclusion that e-cigarettes are less harmful and less addictive, let alone have health benefits. ‘Harm reduction’ claims could be misleading and are likely to be another attempt to sustain a business based on nicotine dependence, which have also been used in promoting ‘safer’ cigarettes and snus^35,36^.

The public should be informed about the updated scientific evidence on the harm and addictiveness of e-cigarettes and counter-marketing should be considered to counteract misleading messages. More stringent regulations, including bans on promotion, flavors, use in smoke-free areas etc., on e-cigarettes should be considered to minimize the potential for smoking renormalization and to maximize their effectiveness on harm reduction. These stronger regulations on e-cigarettes are widely supported by the public, regardless of CC or e-cigarette smoking status^11,16,37,38^.

### Limitations

This study has several limitations. First, the cross-sectional design precludes drawing causal conclusions. Longitudinal surveys at regular intervals to monitor changes in perceptions of e-cigarettes (absolute and relative to CCs) and support for endgame policies on CCs are needed. Second, the current sample may not represent the general Hong Kong population since those without a landline at home or not covered by our sampling frame could not be included. No information on refused respondents could be collected and thus the effect of potential bias could not be estimated, but the data were weighted to the Hong Kong census data to handle oversampling of current and ex-smokers and the difference in age and gender distribution between the current sample and the underlying population. Third, our results may not be generalizable to other countries where the level of TC regulations and acceptability of smoking are different, but may still shed light on future scenarios for public support for TC when these countries tighten TC policies and reduce CC smoking prevalence.

## CONCLUSIONS

Positive perceptions of e-cigarettes relative to CCs were associated with less support for endgame policies on CCs in adults in Hong Kong. Public health actions are needed to disseminate evidence-based knowledge on e-cigarettes. More stringent regulations on e-cigarette promotions and use should be considered and public knowledge of and attitudes towards e-cigarettes should be continuously monitored.
